# Intestinal microbiota development in the first week of life of preterm newborns

**DOI:** 10.1016/j.jped.2025.02.003

**Published:** 2025-04-09

**Authors:** Jessica Santos Passos Costa, Heli Vieira Brandão, Camilla da Cruz Martins, Raquel Guimarães Benevides, Jean Carlos Zambrano Contreras, Luiz Gustavo Sparvoli, Pedro Augusto Ramos Vanzele, Carla Romano Taddei, Tatiana de Oliveira Vieira, Graciete Oliveira Vieira

**Affiliations:** a Universidade Estadual de Feira de Santana (UEFS), Feira de Santana, BA, Brazil; b Universidade Federal do Ceará (UFC), Fortaleza, CE, Brazil; c Universidad de Los Andes, Mérida, Venezuela; d Universidade de São Paulo (USP), São Paulo, SP, Brazil

**Keywords:** Preterm newborn, Microbiota, 16S RRNA

## Abstract

**Objective:**

This study aimed to evaluate the intestinal microbiota development in the first week of life of preterm newborns (PTNB) treated at a public hospital in a municipality in the Brazilian Northeast.

**Methods:**

This is an observational, longitudinal, and descriptive study with 23 PTNBs. Two stool samples were collected from each neonate (fasting/meconium and seventh day of life) for stool microbiota analysis by 16S rRNA gene sequencing. The authors analyzed alpha diversity (Chao1, Shannon, and Simpson indices) and principal coordinates of beta diversity.

**Results:**

Forty-six stool samples from 23 PTNBs were analyzed at the taxonomic level. Microbiota's development was dynamic with low diversity. The authors observed a statistical association with the genera *Enterobacterales, Streptococcus, Bacteroides, Clostridium_sensu_stricto_1, Enterococcus*, and *Bifidobacterium* in the fasting samples when compared to the day-7 samples. The genus *Staphylococcus* also dominated at both times.

**Conclusion:**

Dynamics were observed in the intestinal microbiota development, with an alpha diversity decrease in the stool samples collected at fasting/meconium and on the seventh day of life.

## Introduction

The intestinal microbiota is a microbial ecosystem involved in multiple interactions with the host, such as the delivery type (cesarean section versus vaginal), antibiotics (mother, baby, or both), human milk versus artificial feeding, and the introduction of complementary feeding and weaning.^1^, ^2^, ^3^ As the child grows, the microbiota develops and influences health throughout life until it becomes stable around 18 to 24 months.^4^

Another critical factor in establishing infant intestinal microbiota is gestational age at birth. Studies have shown differences in the stool microbiota of preterm and term newborns.^1^ PTNBs have specific and unique characteristics and face severe health challenges, such as immunological, respiratory, and neurological problems because they are immature. Moreover, they are usually exposed to antibiotics, prolonged hospital stays, use a respirator, and are fed artificially or parenterally. This atypical care environment in the Neonatal Intensive Care Unit (NICU) negatively interferes with the natural pattern of acquisition and development of the healthy intestinal microbiota.^1^, ^2^, ^3^, ^4^

Although the microbiota-host interaction occurs throughout life, it is particularly relevant at birth, when changes in its composition can affect later stages, with an increased risk of several metabolic or immunological disorders.^3^ For this reason, the complex factors involved in establishing the neonatal intestinal microbiota have gained interest in recent years. With this in mind, the current study aims to assess the intestinal microbiota development in the first week of life of PTNBs treated in a public hospital in a municipality in the Brazilian Northeast.

## Methods

### Study characterization

This is a descriptive study, with primary data, of the intestinal microbiota of a group of PTNBs nested in a controlled, non-randomized, superiority clinical trial entitled “*Metagenomic analysis of the intestinal microbiota of preterms undergoing oropharyngeal immunotherapy with colostrum attended at the SUS: an intervention study.*” The clinical trial was approved by the Research Ethics Committee of the State University of Feira de Santana (CAAE N°16995219.0.0000.0053) and the Brazilian Registry of Clinical Trials (UTN: U1111–1248–6732). Mothers of PTNBs were invited to participate in the research within the first 24 h of delivery and supported by the psychology service.

### Sample

The authors included all PTNBs born in 2021 and treated at the State Children's Hospital (HEC) in Feira de Santana (a mid-level metropolitan city in the state of Bahia, Brazil) under the following eligibility criteria: birth weight ≤ 1.500 g, ≤ 36 weeks gestational age, on zero oral and enteral diet or using Total Parenteral Nutrition (TPN) or enteral administration of (pasteurized) human milk from the hospital's Milk Bank. Newborns using vasopressor medication > 10 mg/Kg/min, requiring immediate surgical intervention, and with syndromes or congenital malformations were excluded.

### Stool sample collection

Two samples were collected in the neonatal unit daily from each PTNB in the first week of life; one corresponded to the newborn's first fasting dejection (meconium – T0) and the other on the seventh day of life (T1).

The samples were collected under a specific protocol to preserve existing bacterial species and the quality of the metagenomic DNA. Additional information on the collection of stool samples is available in a published manuscript.^5^

### Variables

The maternal variables surveyed were maternal age, self-reported ethnicity/skin color, marital status, place of residence, parity, number of prenatal visits, delivery type, gestational diabetes, gestational hypertension, smoking, coronavirus infection, urinary infection, chronic kidney disease, and maternal syphilis.

The variables relating to premature babies were: a) Clinical data - sex, gestational age, birth weight, use of antibiotics, broad-spectrum antibiotic, oxygen therapy type, umbilical catheter, central venous access, peripherally inserted central catheter, abdominal distension, gastric residue, mucosanguineous stools, regurgitation; b) Morbidity and mortality data - death, intraventricular hemorrhage, renal failure, neonatal sepsis, patent ductus arteriosus, pneumonia, pneumothorax, hyaline membrane disease (HMD), and c) Nutritional data - time to start an enteral diet, parenteral nutrition time, weight on the seventh day of life, and type of diet on the 7th day of life. The information about the newborn was recorded on a specific spreadsheet.

### DNA extraction

The stool samples’ total DNA was extracted using the QIAamp PowerFecal Pro DNA Kit (QIAGEN, Hilden, Germany). This protocol involves using 250 mg of stool for cell lysis, employing beads and a lysis solution in a TissueLyser II (QIAGEN, Hilden, Germany). The lysis is achieved by high-speed shaking at an oscillation frequency of 25 Hz for 10 min. The following steps were performed according to the manufacturer's standards. The extracted DNA was then eluted in 80 µL of DNase/RNase-free sterile water. After extraction, the DNA from the stool samples was measured using the Qubit Fluorometer (Thermo Fisher Scientific, Waltham, USA) using the Qubit™ dsDNA BR Assay kit, and then stored at −80 °C until the Polymerase Chain Reaction (PCR) amplification stage.

### Sample sequencing

Amplification of the V3-V4 region of the 16S rRNA gene.

The newborns’ stool microbiota was characterized by amplifying the V3-V4 region of the bacterial 16S ribosomal gene. The primer sequences used for this region were V3-V4 forward primer and V3-V4 reverse primer, described by Klindworth et al.^11^, with Illumina adapters. The target sequences were amplified with 5 μL of microbial DNA (10ng/μL) in a total volume of 25 μL, also consisting of 5 μL of each primer, 2.5 μL of AccuPrime PCR Buffer II (ThermoFisher), 0.2 μL of AccuPrime Taq DNA Polymerase (ThermoFisher), and 7.3 μL of DNase/RNase-free sterile water. The reaction was performed under the following conditions: an initial cycle of 94 °C for 2 min, followed by 30 cycles consisting of denaturation at 94 °C for 30 s, annealing at 55 °C for 30 s, extension at 68 °C for 45 s, and a final cycle of 68 °C for 2 min. The amplicon size after the PCR step is approximately 550 bp.

The amplicons from the PCR step were subjected to an indexing PCR using two adapters from the Nextera XT Index Kit Set A. Each reaction contained 5 μL of Nextera XT Index 1 Primers (N7XX) and 5 μL of Nextera XT Index 2 Primers (N7XX), besides 5 μL of the PCR amplicon, 5 μL of AccuPrime PCR Buffer II (ThermoFisher), 1.3 μL of AccuPrime Taq DNA Polymerase (ThermoFisher), and 28.7 μL of DNase/RNase-free sterile water, in a final volume of 50 μL. The reaction includes an initial cycle at 94 °C for 2 min, followed by 8 cycles of 94 °C for 30 s, 55 °C for 30 s, and 68 °C for 45 s, with a final cycle of 68 °C for 2 min. After the indexing step, the target fragment size was approximately 630 bp. The amplicons were then quantified and normalized to a concentration of 4 nM.

For sequencing, the amplicons were pooled and loaded onto Illumina MiSeq clamshell style cartridge kit V2 (500 cycles), for paired-end 250 sequencing, at a final concentration of 8 pM. The library was clustered to a density of approximately 820 k/mm^2^. All procedures were carried out following the manufacturer's protocol (Illumina-16S Metagenomic Sequencing Library Preparation).^6^

### Microbiota analysis using bioinformatics tools

After obtaining the sequences, the 16S rRNA libraries were analyzed using the QIIME v.2-2020.2 software.^7^ Denoising was performed through the DADA2 tool.^8^ The direct sequences were then truncated at position 251 nucleotides, while the reverse sequences were truncated at 250 nucleotides to discard the positions for which the median nucleotide quality was lower than Q30. Samples with <1000 sequences were also excluded from further analysis.

Taxonomy was assigned using ASVs (Amplicon Sequencing Variant) via the q2-feature classifier resource and the Bayes naive taxonomy classifier classifysklearn, comparing the ASVs obtained against the SILVA 132 reference database.^9^^,^^10^ The subsequent analyses were carried out in SPSS software version 26 and R version 4.2.2, using the phyloseq, vegan, microbiome, and ggplot2 packages.^11^, ^12^, ^13^, ^14^

### Statistical analysis

The analyses were conducted using SPSS version 26 and R version 4.2.2. The Chao1 richness index, Shannon diversity index, and Simpson diversity index were evaluated for the alpha diversity analysis. Besides the beta diversity analysis, the authors also evaluated the difference in the 15 most abundant bacterial genera in the stool samples. The effect of time on the intestinal microbiota was assessed in all the analyses, comparing between the different periods.

Descriptive measures such as mean and standard deviation for numerical variables and absolute and relative frequencies for categorical variables were calculated. The adherence to normality was first assessed using the Shapiro-Wilk test to check for variations over time. Next, the non-parametric Wilcoxon rank sum exact test was adopted, similar to the Student's *t*-test for two related samples. A significance level of *p* < 0.05 was employed.

The alpha diversity indices (Chao1, Shannon, Simpson) were calculated using Generalized Estimating Equations (GEE). The models were evaluated using gamma or linear distributions and the identity link function. The correlation matrix varied between independent, AR, unstructured, and exchangeable. The lowest quasi-likelihood under the Independence Criterion (QIC) value was considered to select the best model. The best adherence of the residuals was also assessed using the Q-Q plot.^15^

In the beta diversity analysis, the PERMANOVA test was performed for each variable with the adonis2 function (vegan package), using the weighted and unweighted UniFrac distances. Nine hundred ninety-nine permutations were made for each variable. A p-value < 0.05 was considered statistically significant.

The authors performed the Principal Coordinate Analysis (PCoA), a graphical representation that allows multidimensional data to be analyzed on a two-dimensional plane.

## Results

Eighty stool samples were collected from 40 PTNBs for the intestinal microbiota analysis. After bioinformatic analysis, 34 samples were excluded (17 infants) because they had low DNA read counts (< 1000 reads). Forty-six samples from 23 newborns were analyzed and sequenced. The descriptive characteristics of the mothers, control PTNBs, and excluded PTNBs in the study are shown in Table 1; and, it is noteworthy that there were no discrepant differences between the compared groups.

**Table 1:** Descriptive statistics of mothers and their premature newborns in the first week of life, 2023.

Variables	RN Control	RN Excluded		
	N (%)	N(%)		
Maternal age	**23**	**15**		
≥ 18 years	21 (91.3)	14 (93.3)		
< 18 years	2 (8.7)	1 (6.7)		
Self-declared ethnicity/skin color	**23**	**17**		
White	1 (4.3)	3 (17.6)		
Non-white	22 (95.7)	14 (82.4)		
Marital status	**20**	**16**		
With partner	11 (55)	8 (50.0)		
Without partner	9 (45)	8 (50.0)		
Place of residence	**23**	**17**		
Urban	18 (78.3)	10 (58.8)		
Rural	5 (21.7)	7 (41.2)		
Parity	**18**	**17**		
Multiparous	9 (50)	13 (76.5)		
Primiparous	9 (50)	4 (23.5)		
Number of prenatal care visits	**16**	**14**		
≥ 6 visits	4 (25)	9 (64.3)		
< 6 visits	12 (75)	5 (35.7)		
Delivery type	**23**	**17**		
Vaginal	11 (47.8)	11 (64.7)		
Cesarean	12 (52.2)	6 (35.3)		
Gestational diabetes	**23**	**16**		
No	21 (91.3)	13 (81.3)		
Yes	2 (8.7)	3 (18.8)		
Gestational hypertension	**23**	**16**		
No	17 (74)	10 (62.5)		
Yes	6 (26)	6 (37.5)		
Smoker	**23**	**15**		
No	22 (91.3)	15 (100.0)		
Yes	1 (4.4)	0 (0.0)		
Coronavirus infection	**23**	**17**		
No	22 (95.6)	17 (100.0)		
Yes	1 (4.4)	0 (0.0)		
Urinary infection	**23**	**16**		
No	20 (86.9)	11 (68.75)		
Yes	3 (13.1)	5 (31.25)		
Chronic kidney disease	23	17		
No	22 (95.6)	16 (94.12)		
Yes	1 (4.4)	1 (5.88)		
Maternal syphilis	**23**	**17**		
No	22 (95.6)	17 (100.0)		
Yes	1 (4.4)	0 (0.0)		

Descriptive statistics of preterm newborns in the first week of life				
Variables	RN Control Mean ± Standard Deviation	RN Excluded Mean ± Standard Deviation	RN Control N ( %)	RN Excluded N ( %)
Clinical data				
Newborn sex			**23**	**17**
Female	–	–	12 (52.2)	7 (41.2)
Male	–	–	11 (47.8)	10 (58.8)
Gestational age			**23**	**15**
≥ 28 weeks	–	–	13 (56.6)	9 (60.0)
< 28 weeks	–	–	10 (43.5)	6 (40.0)
Gestational age (weeks)	29.09 ± 2.6	28.13 ± 2.7	–	–
Birth weight (grams)	1055.2 ± 224.2	1074.59 ± 294.82	–	–
Birth weight			**23**	**17**
≤ 1500 > 1000 g (VLBW)^a^	-	–	12 (52.2)	11 (64.7)
< 1000 g (ELBW)^a^	-	–	11 (47.8)	6 (35.3)
Use of antibiotics			**23**	**17**
No	–	–	2 (8.7)	0 (0.0)
Yes	–	–	21 (91.3)	17 (100.0)
Broad-spectrum antibiotic			**21**	**15**
Ampicillin/Gentamicin/Oxacillin/Amikacin	–	–	14 (66.7)	10 (66.7)
Piperacillin/Tazobactan/Vancomycin/Meropenem	–	–	7 (33.3)	5 (33.3)
Oxygen Therapy			**23**	**17**
Non-invasive	–	–	9 (39.1)	3 (17.6)
Invasive	–	–	14 (60.9)	14 (82.4)
Umbilical catheter			**23**	**17**
No	–	–	0 (0.0)	0 (0.0)
Yes	–	–	23 (100.0)	17 (100.0)
Central venous access			**23**	**17**
No	–	–	20 (87.0)	14 (82.3)
Yes	–	–	3 (13.0)	3 (17.7)
Peripherally Inserted Central Catheter			**23**	**16**
No	–	–	8 (34.8)	9 (52.9)
Yes	–	–	15 (65.2)	8 (47.1)
Abdominal distension			**23**	**17**
No	–	–	11 (47.8)	9 (52.9)
Yes	–	–	12 (52.2)	8 (47.1)
Gastric residue			**23**	**17**
No	–	–	7 (30.5)	6 (35.3)
Yes	–	–	16 (69.5)	11 (64.7)
Mucosanguineous stools			**23**	**17**
No	–	–	22 (95.7)	17 (100.0)
Yes	–	–	1 (4.3)	0 (0.0)
Regurgitation			**23**	**17**
No	–	–	11 (47.8)	11 (64.7)
Yes	–	–	12 (52.2)	6 (35.3)

Morbidity and mortality data	Mean ± Standard Deviation	Mean ± Standard Deviation	RN Control N ( %)	RN Excluded N ( %)
Death			**23**	**17**
No	–	–	20 (87.0)	15 (88.2)
Yes	–	–	3 (13.0)	2 (11.8)
Intraventricular hemorrhage			**23**	**17**
No	–	–	21 (91.3)	14 (82.4)
Yes	–	–	2 (8.7)	3 (17.6)
Renal Failure			**23**	**16**
No	–	–	21 (91.3)	14 (87.5)
Yes	–	–	2 (8.7)	2 (12.5)
Neonatal sepsis			**23**	**17**
No	–	–	2 (8.7)	0 (0.0)
Yes	–	–	21 (91.3)	17 (100.0)
Patent ductus arteriosus			**23**	**17**
No	–	–	21 (91.3)	16 (94.1)
Yes	–	–	2 (8.7)	1 (5.9)
Pneumonia			**23**	**17**
No	–	–	22 (95.7)	17 (100.0)
Yes	–	–	1 (4.3)	0 (0.0)
Pneumothorax			**23**	**17**
No	–	–	23 (100.0)	17 (100.0)
Yes	–	–	0 (0.0)	0 (0.0)
Hyaline Membrane Disease			**23**	**17**
No	–	–	19 (82.7)	6 (35.3)
Yes	–	–	4 (17.3)	11 (64.7)

Nutritional data	Mean ± Standard Deviation	Mean ± Standard Deviation	RN Control N ( %)	RN Excluded N ( %)
Time to start an enteral diet (days)	1.66 ± 1.45	1.94 ± 1.34	–	–
Parenteral nutrition time (days)	6.04 ± 1.63	5.25 ± 2.2	–	–
Weight on day 7 (grams)	1010.9 ± 208.7	1570.1 ± 2263.5	–	–
Type of diet on the 7th day of life	–	–	**19**	**13**
Fast	–	–	4 (21.1)	0 (0.0)
Exclusive breast milk	–	–	15 (78.9)	13 (100.0)
Breast milk + formula	–	–	0 (0.0)	0 (0.0)

a VLBW, Very low birth weight; ELBW, Extremely low birth weight.

### Alpha diversity and beta diversity

**Figure 1:**
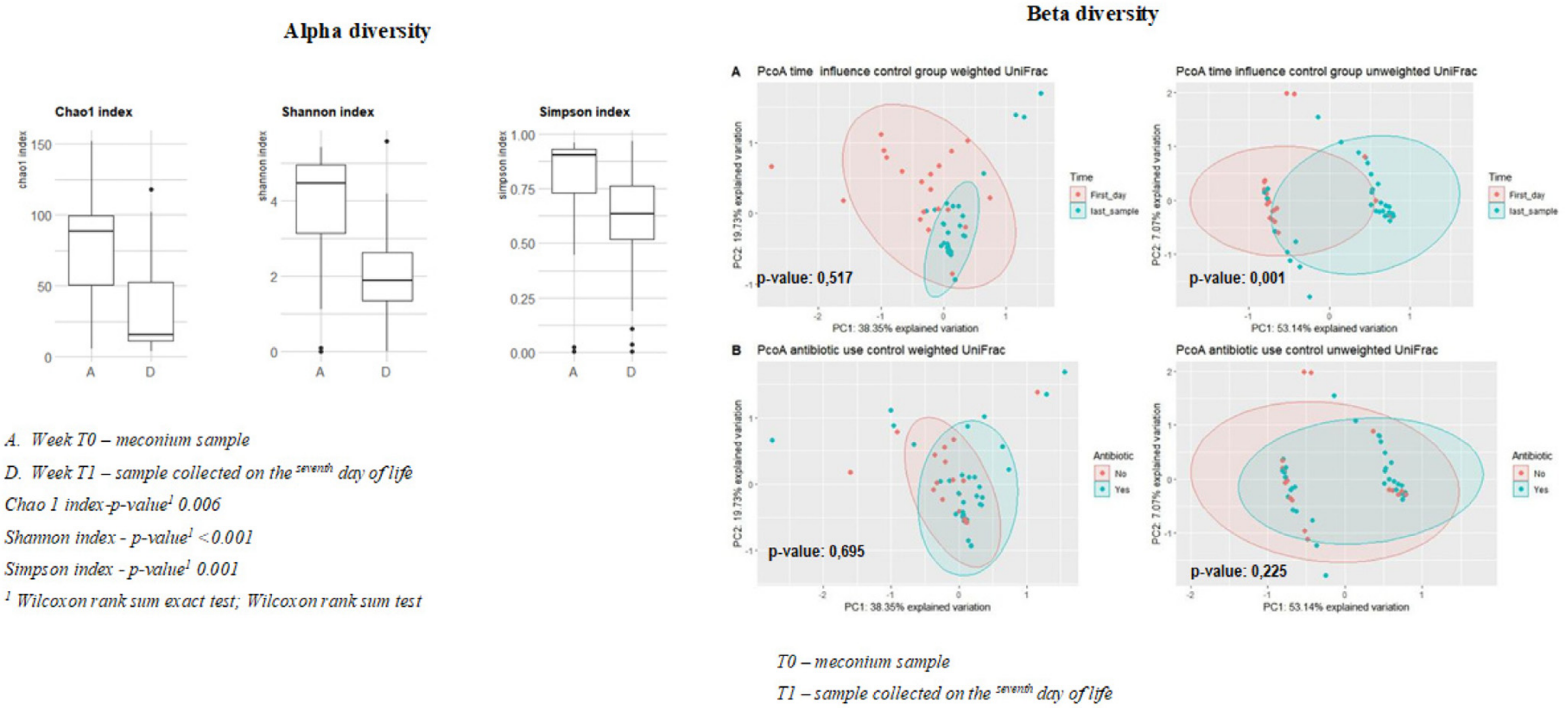
Chao 1, Shannon, and Simpson diversity indices in preterm newborns' first week of life, and the beta diversity principal coordinates analysis, comparisons over the first week T0 and T1 and antibiotic use, 2023.

The results of the alpha diversity indices (Chao1, Shannon, and Simpson) regarding time (T0 – first sample collected / T1 – sample collected on the seventh day of life) are shown in Figure 1. The Shannon diversity index shows a significant reduction in microbial diversity when comparing T0 (first sample collected) with T1 (sample collected on the seventh day of life) (4.46 vs. 1.88; *p* < 0.001). Simpson's diversity index ranges from 0 to 1 and measures the probability that two individuals taken randomly from the community belong to the same species; 0 (zero) represents no diversity, and 1 infinity diversity. The results indicate statistically significant differences in Simpson's index at T0 compared to T1 (0.90 vs. 0.63; *p* = 0.001) (Figure 1). Analysis of the samples between the first collection and the last collection (after the enteral diet had started) showed a downward trend in alpha diversity (Shannon 4.46 vs. 1.88; Chao1 76.7 vs. 36.9; Simpson 0.90 vs. 0.63), although biological diversity was found in all the tests.

The differences in beta diversity can be observed using a Principal Coordinates Analysis (PCoA) plot based on the weighted and unweighted UniFrac distance matrices. The coordinate analysis considered two groups, the first by collection time between samples (T0 and T1) and prophylactic antibiotics (yes and no). There was no statistical significance in the weighted analysis between T0 and T1 (*F* = 0.77; *P* = 0.51) nor regarding the use of antibiotics (*F* = 0.54; *P* = 0.69). In the unweighted analysis, there was significance only in terms of the time between samples (*F* = 8.92; *P* = 0.001), which was not found for antibiotic use (*F* = 1.33; *P* = 0.22) (Figure 1).

### Genera relative abundance

**Figure 2:**
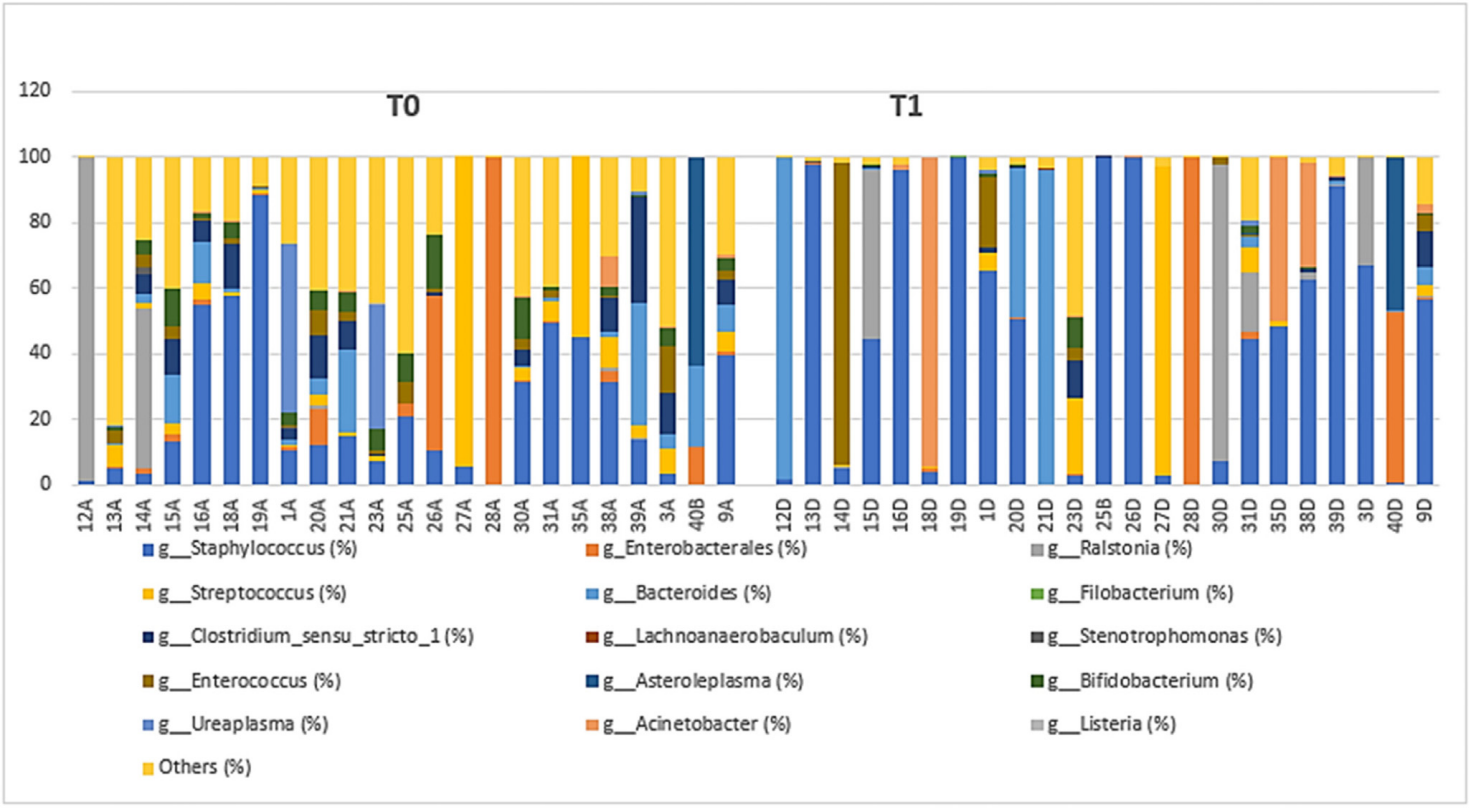
Relative genera abundance in stool samples from preterm newborns over time, 2023. T0 – First fasting sample–meconium T1–Sample on the 7th day of life.

Statistical analysis and the distribution of the 15 most abundant bacterial genera in the stool samples at T0 and T1 were performed, described in Table 2 and Figure 2. The relative abundance of the most prevalent bacterial genera in the samples shows the dominance of three taxa observed in Table 2.

**Table 2:** Composition and taxonomic variations of samples at genus level and their relative abundance over time, 2023.

Genus	Week		*p*-value^b^
	T0^a^ (%)	T1^a^ (%)	
*g_Staphylococcus*	**22.57**	**45.59**	0.11
*o_Enterobacterales*	**8.10**	**6.85**	**0.041**
*g_Ralstonia*	6.5	**8.57**	0.7
*g_Streptococcus*	**9.18**	5.59	**0.019**
*g_Bacteroides*	6.18	**10.90**	**0.022**
*g_Filobacterium*	0.0047	0.0026	0.9
*g_Clostridium_sensu_stricto_1*	5.77	1.21	**0.038**
*g_Lachnoanaerobaculum*	0.0026	0.0040	0.5
*g_Stenotrophomonas*	0.103	0.0035	0.2
*g_Enterococcus*	2.54	5.46	**0.010**
*g_Asteroleplasma*	2.77	2.05	0.7
*g_Bifidobacterium*	4.16	0.62	**<0.001**
*g_Ureaplasma*	3.98	0.10	0.3
*g_Acinetobacter*	0.55	7.87	0.6
*g_Listeria*	0.0056	0.000	0.081
*Others*	27.46	4.77	**<0.001**

a Week T0 – meconium sample/Week T1 – sample collected on the 7th day of life.

b *p* < 0.05.

After statistical analysis, the analysis of composition and taxonomic variations showed statistical significance (*p* < 0.05) in T0 against T1 for taxa. No statistically significant differences were identified in the relative abundance of the other eight genera tested (Table 2).

## Discussion

The current study aimed to describe the intestinal microbiota's development and diversity in two different stages: birth, based on the analysis of meconium, and on the seventh day of life from 23 PTNBs.

Analyzing the Chao1, Shannon, and Simpson indices allowed us to estimate the patterns of richness and diversity of the microbial community of the intestinal microbiota of preterms, and the authors observed a decrease in alpha diversity in the stool samples collected between T0 and T1, characteristic has been observed in other studies and is considered a dysbiosis marker.^16^

As for beta diversity, the authors observed significant differences in the unweighted analysis between the samples (T0/T1), which shows a change in the composition of the microbial communities over time. In the meconium, the authors found a higher relative abundance of the taxa *Staphylococcus, Streptococcus*, and *Enterobacterales. Staphylococcus, Bacteroides, Ralstonia*, and Enterobacterales were more abundant on the seventh day of life.

However, when the taxonomic variations were analyzed at the two collection stages, a significant decrease was observed in *Enterobacterales, Streptococcus, Clostridium_sensu_stricto_1*, and *Bifidobacterium*, and an increase in the genera *Bacteroides, Enterococcus, Staphylococcus*, and *Acinetobacter*, although only the first two were statistically significant. Thus, the authors observed that the bacterial community may be being maintained by all the bacteria present, regardless of their abundance, as a whole, and not just by the prevalent group.

In all the measurements (alpha, beta diversity, and relative abundance), the authors observed that babies’ microbial communities become more homogeneous at T1 when abundance (weighted) is considered, although this was not significant. There is also an apparent change in the composition of the species at the different stages, a decreased diversity (significant reduction in “Others” and decline in Chao1), and a significant difference in unweighted beta (which only considers the presence/absence of microorganisms). Some factors are cited in the literature as contributing to these changes, such as the colonization and establishment in the first days of life, the implementation of enteral feeding, the acquisition of microorganisms from the hospital environment, and the high prevalence of antibiotic use in the groups studied.^1^^,^^16^

The diversity of intestinal microbiota at both stages is expected since the meconium microbiota mainly reflects prenatal and neonatal factors.^16^, ^17^, ^18^, ^19^ Previous maternal infections, such as those observed in this study, syphilis (baby number 15), urinary infection (babies numbers 12, 15, and 27), coronavirus infection (baby number 27), and gestational diabetes (babies numbers 15 and 28) may have influenced the newborns’ colonization profile.

The intestinal microbiota on day 7 reflects the newborns’ exposure to the extrauterine environment. The gut microbiota is characterized by low diversity and high inter-individual variability in very premature newborns, which can be attributed to several conditions, such as cesarean delivery, prolonged exposure to the environment, and neonatal intensive care unit (NICU) practices, involving isolation in incubators, oxygen use, intubation, extubation, and the use of broad-spectrum antibiotics.^16^ Also, prematurity and diet influence the dynamics of intestinal bacterial establishment.^1^

The present study identified a high prevalence of anaerobic bacteria such as *Staphylococcus* and *Streptococcus* in the samples. Similarly, a study conducted in Indonesia, showed decreasing diversity and complexity of the microbiome when comparing stool samples in the meconium on the fourth and seventh days of life.^20^

The authors identified an increased prevalence of *Bacteroides* over time (T0/T1). The upward trend of this genus at the end of the first week of the PTNB's life may reflect the type of delivery, which is generally one of the main factors determining initial colonization since *Bacteroides* characterize the normal vaginal microbiome.^2^^,^^21^ Vaginal delivery was observed in almost half of the PTNB mothers evaluated. Moreover, a more anaerobic environment can also help to establish *Bacteroides*.^19^^,^^21^

The evaluated meconium samples were derived from PTNBs on a zero diet. The stool seventh-day samples, on the other hand, were influenced by the type of feeding and the time when the enteral diet was started via an orogastric tube with human milk from the human milk bank (HMB), which helps with food tolerance and intestinal health, although it has a different impact on the baby's intestinal microbiota when compared to the mother's raw milk. However, both have a marked influence on the stool microbiota when compared to the microbiota of those who use formula.^22^^,^^23^ The differences in intestinal microbial composition between breastfed and formula-fed babies are well documented, with higher bifidobacteria levels in those fed with human milk.^1^^,^^24^ In this sense, considering that all the PTNBs in the current study were exclusively consuming human milk on day 7, this microbiota was expected to show a greater abundance of *Bifidobacterium*. However, the authors found a decline in the mean prevalence in (T1).

The literature shows that PTNBs show delayed intestinal colonization with commensal anaerobic species such as *Bifidobacterium* or *Bacteroides*, where instead their stools contain significantly higher *Enterobacteriaceae, Enterococcus*, and *Enterobacterales* levels.^1^^,^^22^^,^^23^ Another factor that needs to be considered in the cohort is the early collection of stool samples, which may not have allowed the genus *Bifidobacterium* to reach a state of dominance that would allow it to be evidenced since the alpha diversity of the intestinal microbiota in PTNBs increases as preterms age.^25^^,^ ^26^

Similarly, a study conducted in Indonesia found a low *Bifidobacterium* and *Lactobacillus* prevalence, attributed to the mother's diet, which was low in dairy products.^18^ Other possibilities that determine the low *Bifidobacterium* prevalence are exclusive feeding of human milk from the milk bank, which has a varied composition of bioactive components (all the newborns were on it) and antibiotic use (adopted by a large proportion of the babies).^1^^,^^26^ Furthermore, the delay in starting the enteral diet, which was approximately one and a half days for the newborns in this study, may also have contributed to the low concentration of *Bifidobacterium*. In very low and extremely low birth weight PTNBs, the start of the diet is delayed due to characteristics of prematurity, such as immaturity of the digestive system and clinical instability.^1^

Although there was no statistical significance regarding the genus *Staphylococcus* in this trial, the authors observed a high prevalence of relative abundance in both groups (T0 and T1), corroborating other studies that have pointed to the dominance of this genus in the meconium of PTNBs, especially in cesarean births.^19^^,^^27^ The high abundance of these bacteria may have contributed to neonatal sepsis.^28^ The increase in *Staphylococcus* was also found in another study.^29^ It can be explained by the bacterial transfer from human milk to the PTNB and the swallowing of bacteria in the oral cavity that have not adhered to the mucosa and participate in intestinal colonization.^29^

Furthermore, the authors observed a higher *Clostridium sensu stricto 1* prevalence in the meconium samples against the seventh day. The *Clostridium sensu stricto 1* genus includes >20 species, some of which have pathogenic potential, and others have commensal characteristics.^30^ PTNBs born by cesarean section, the prevailing delivery type in the current study, have a reduced complexity of intestinal microbiota and are more frequently colonized by the genera *Clostridium sensu stricto 1* and *Clostridium difficile*, by environmental microorganisms from the mother's skin, unlike those born vaginally, who result in gut colonization by microorganisms associated with the vagina such as *Bifidobacterium* and *Bacteroides* because they come into contact with the maternal vaginal and fecal microbiota. A study demonstrated that the intestinal microbiota of preterm infants reflects the diverse vaginal microbiota.^21^

Factors such as human milk feeding may have possibly contributed to correcting this sign of intestinal dysbiosis identified in the meconium samples.^1^ A cohort study conducted with 1249 mother-baby dyads provided evidence that human milk can transfer bacteria to the newborn's intestine and influence the development of the intestinal microbiota to an extent similar to other infant microbiome modifiers, such as the birth type.^8^

These results reflect the findings of the intestinal microbiota of a group of PTNBs admitted to the NICU of a city in the Brazilian Northeast. The authors noticed that the neonates’ intestinal microbiota development was dynamic and with low diversity, with variations in the following genera: *Enterobacterales, Streptococcus, Bacteroides, Clostridium_sensu_stricto_1, Enterococcus* and *Bifidobacterium*. The genus *Staphylococcus* prevailed in both stages.

As limitations, the authors highlight: the short follow-up time of the PTNB, the use of prophylactic antibiotic therapy and the failure to carry out a comparative analysis of specific populations, such as subgroups of newborns born small for gestational age and extremely premature infants. Furthermore, the convenience sample and small sample size may have affected the study's statistical power, hindering the generalization of the results to all PTNBs or full-term births.

The strengths of the present study include its relevance in research on the intestinal microbiota development in the first week of life of preterm newborns, initially on a zero diet and fed with human milk from the HMB via an orogastric tube until the seventh day of life. Furthermore, the careful stool sample collecting technique avoids contamination and allows the evaluation of the 16S rRNA gene by metagenomic analysis.

## Funding/support

Conselho Nacional de Desenvolvimento Científico e Tecnológico. Edital Universal CNPq 28/2018.

## Role of funder/sponsor (if any)

Conselho Nacional de Desenvolvimento Científico e Tecnológico (CNPq) had no role in the design and conduct of the study.

## Authors’ contributions

All authors approved the final manuscript as submitted and agreed to be accountable for all aspects of the work.

## Trial registration

*World Health Organization* (WHO) under Universal Trial Number (UTN) code U1111-1266-2295, under register RBR-3mm7cs in the Brazilian Registry of Clinical Trials (REBEC).

## Conflicts of interest

The authors declare no conflicts of interest.
